# Organ dysfunction, injury, and failure in cardiogenic shock

**DOI:** 10.1186/s40560-023-00676-1

**Published:** 2023-06-29

**Authors:** Akihiro Shirakabe, Masato Matsushita, Yusaku Shibata, Shota Shighihara, Suguru Nishigoori, Tomofumi Sawatani, Kazutaka Kiuchi, Kuniya Asai

**Affiliations:** 1 Division of Intensive Care Unit, Nippon Medical School Chiba Hokusoh Hospital, 1715 Kamagari, Inzai, Chiba 270-1694 Japan; 2 Department of Cardiovascular Medicine, Nippon Medical School, Tokyo, Japan

**Keywords:** Acute decompensated heart failure, Biomarker, Mortality, Inflammation

## Abstract

**Background:**

Cardiogenic shock (CS) is caused by primary cardiac dysfunction and induced by various and heterogeneous diseases (e.g., acute impairment of cardiac performance, or acute or chronic impairment of cardiac performance).

**Main body:**

Although a low cardiac index is a common finding in patients with CS, the ventricular preload, pulmonary capillary wedge pressure, central venous pressure, and systemic vascular resistance might vary between patients. Organ dysfunction has traditionally been attributed to the hypoperfusion of the organ due to either progressive impairment of the cardiac output or intravascular volume depletion secondary to CS. However, research attention has recently shifted from this cardiac output (“forward failure”) to venous congestion (“backward failure”) as the most important hemodynamic determinant. Both hypoperfusion and/or venous congestion by CS could lead to injury, impairment, and failure of target organs (i.e., heart, lungs, kidney, liver, intestines, brain); these effects are associated with an increased mortality rate. Treatment strategies for the prevention, reduction, and reversal of organ injury are warranted to improve morbidity in these patients. The present review summarizes recent data regarding organ dysfunction, injury, and failure.

**Conclusions:**

Early identification and treatment of organ dysfunction, along with hemodynamic stabilization, are key components of the management of patients with CS.

## Background

Shock is a manifestation of acute circulatory failure, in which the circulatory system fails to provide cells/tissues with sufficient oxygenated blood to optimally perform their functions [1]. Cardiogenic shock (CS) is caused by primary cardiac dysfunction, resulting in an inadequate cardiac output due to multiple conditions. These conditions include acute impairment of cardiac performance (e.g., acute coronary syndrome [ACS] and acute myocarditis) or acute or chronic impairment of cardiac performance (exacerbation of chronic decompensated heart failure [HF] and/or natural progression of advanced HF). Several decades ago, CS was described as a status of hypoperfusion due to a significant reduction in cardiac index (CI), leading to peripheral vasoconstriction and increased pulmonary capillary wedge pressure (PCWP) [2].

CS is mainly caused by ACS and HF etiologies. Its incidence is approximately 6–13% in ACS [3, 4] and 4% in acute HF (AHF) [5–7]. The etiological spectrum of CS is also broad and may include numerous other etiologies, such as ischemic cardiomyopathy without acute myocardial infarction (AMI), non-ischemic cardiomyopathy, incessant ventricular arrythmia, and severe valvular disease [6]. Regardless of the etiology, CS is characterized by inadequate cardiac output leading to hypotension and signs and/or symptoms of end-organ hypoperfusion, such as inadequate renal perfusion (i.e., oliguria) [8]. It is also characterized by the biochemical manifestations of hypoperfusion (e.g., elevated levels of serum creatinine and aminotransferases, extremely elevated levels of plasma B-type natriuretic peptide [BNP], metabolic acidosis, and elevated levels of serum lactate). Moreover, it reflects tissue hypoxia and alterations in cellular metabolism, leading to organ dysfunction. Hypoperfusion by CS could lead to injury, impairment, and failure of affected organs (i.e., heart, lungs, kidney, liver, intestines, brain), which was so-called “forward failure”. Meanwhile, venous congestion due to elevation of the right atrial pressure and high venous pressure by CS could lead to congestive organ damages (kidney, liver and intestines), which was so-called “backward failure”. These effects are associated with an increased mortality rate.

**Fig. 1:**
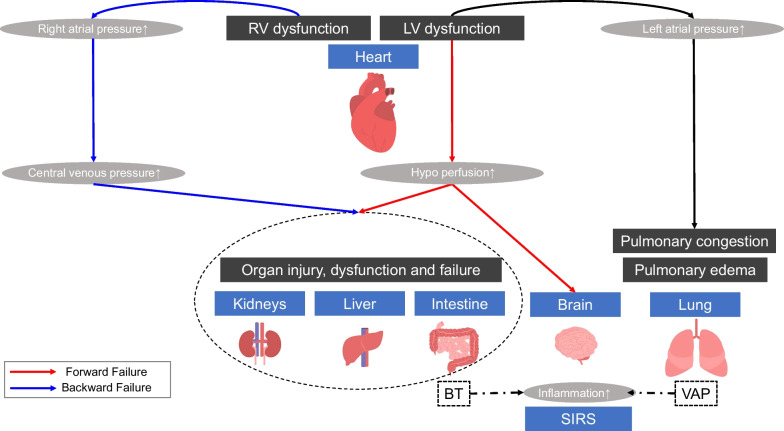
Pathophysiologic cycle in cardiogenic shock (CS). *RV* right ventricular, *LV* left ventricular, *BT* bacterial translocation, *VAP* ventilator-associated pneumonia, *SIRS* systemic inflammatory response

Vital organ dysfunction is thought to be directly linked to poor prognosis in patients with CS. In a recent retrospective analysis covering 444,253 patients with AMI-CS, there was an association between the number of dysfunctional organs and in-hospital mortality, as well as a lower probability of discharge [9]. Both macrohemodynamic alterations and microcirculatory dysfunction have been associated with multi-organ dysfunction; however, the pathophysiology of organs in CS remains unclear [10]. Treatment strategies for the prevention, reduction, and reversal of organ injury are warranted to improve morbidity in these patients. Early identification and treatment of organ dysfunction, along with hemodynamic stabilization, are key components of the management of patients with CS. The present review summarizes recent data regarding organ dysfunction, injury, and failure (Fig. 1; Table 1).

**Table 1:** Assessment and treatment of organ injury in cardiogenic shock (CS)

Organ	Biochemical	Imaging	Monitoring	Mechanical Support
Heart	Natriuretic peptides	Echocardiography	Heart rate and blood pressure	VA-ECMO
	Cardiac troponin	ECG	Central venous catheter	IABP
	Lactate	Ultrasound of IVC	Arterial line and PAC	Impella®
Lungs	Peripheral arterial oxygen saturation	Chest X-ray	PAC	ETI
	arterial blood gas	CT		NIV
		Lung ultrasound		HFNC
Kidneys	Serum creatinine and blood urea nitrogen	Ultrasound	Urinary output	CRRT
	Potassium			
	Arterial blood gas acid balance			
Liver	Transaminases	Ultrasound		
	Bilirubin			
	Albumin			
Intestines	N/A	CT		
Brain	N/A	CT	GCS, CAM–ICU, EEG	TTM

VA-ECMO, venoarterial extracorporeal membrane oxygenation; IABP, intra-aortic balloon pump; ECG, electrocardiography; IVC, inferior vena cava; PAC, pulmonary artery catheter; ETI, endotracheal intubation; CT, computed tomography; NIV, non-invasive positive pressure ventilation; HFNC, high-flow nasal cannula; CRRT, continuous renal replacement therapy; N/A, not applicable; GCS, Glasgow Coma Scale; CAM-ICU, confusion assessment method for the intensive care unit; EEG, electroencephalography; TTM, target temperature management

## Organ systems and organ-specific management options

### Heart

Improvement in oxygen delivery to the end organs is the top priority in CS. Moreover, protection of the primary organ (i.e., the heart) should be simultaneously considered. CS typically develops due to cardiac dysfunction. In CS, the ventricular wall stress and valvular regurgitation increase, while myocardial stretching, myocardial remodeling, and ventricular myocyte necrosis progress by increasing the preload due to congestion. These effects eventually lead to a progressive exacerbation of cardiac dysfunction. Furthermore, cardiac function is further impaired by the decline in coronary flow and increase in the levels of chemical mediators, such as tumor necrosis factor-α (TNF-α) and interleukin-6 (IL-6). In addition, the decline of myocardial compliance in CS leads to the downregulation of β receptor expression. Total ventricular unloading, which is a strategy for preventing the deterioration of cardiac performance, might be achieved by appropriate use of mechanical support in CS.

Current guidelines for AHF suggest the use of short-term mechanical circulatory support in patients with CS, in particular those with persistent unstable hemodynamics and insufficient end-organ perfusion despite medical therapy [11]. An intra-aortic balloon pump (IABP) is the most widely used device for mechanical hemodynamic support in ACS-CS [12]. However, the results of a recent trial showed that its use did not improve outcomes in patients with AMI-CS [13], and it is, therefore, not recommended for routine use in CS. A device selection strategy for venoarterial extracorporeal membrane oxygenation (VA-ECMO), as well as a recommendation for the use of a percutaneous left ventricular assist device (Impella^®^; Abiomed, Inc., Danvers, Massachusetts, USA) for total ventricular unloading in CS, are outlined in the European Society of Cardiology guidelines for HF. The use of Impella 2.5 in CS was established during the past decade. It was expected that immediate initiation of Impella use from the acute phase of CS would result in a marked decrease in PCWP (i.e., decrease in left ventricular filling pressure), reduction in infarct size, and prevention of subsequent HF [14–16]. It was also reported that the combined use of Impella and VA-ECMO was associated with both right (i.e., lower pulmonary artery pulsatility index and central venous pressure [CVP]) and left ventricular unloading effects. This approach reduced myocardial damage and increased the total mechanical circulatory support flow compared with VA-ECMO alone [14]. However, it was recently reported that, compared with VA-ECMO support alone, the combination of Impella and VA-ECMO in patients with CS was associated with an increased rate of complications, such as bleeding, need for renal replacement therapy, hemolysis, and limb ischemia [17]. It remains controversial whether the reduction of ventricular overload with Impella in patients treated with VA-ECMO reduces the rate of early mortality. Further investigation is warranted to test this assumption.

### Lungs

Respiratory failure is present in almost all patients with CS. Although a low CI is a common finding in patients with CS, the ventricular preload such as PCWP, CVP, and after-load such as systemic vascular resistance might vary between patients. CS caused by left ventricular failure might generally present as ‘wet and cold’ based on the Nohria–Stevenson criteria [18], and is classified as hypoperfusion with congestion (low CI, high systemic vascular resistance, CVP, and PCWP). In the SHOCK trial, two-thirds of patients with CS clinically presented with hypoperfusion and congestion [19]. The mechanism underlying the development of lung congestion in patients with CS involved an increase in hydrostatic left arterial pressure and mitral regurgitation. These effects increased the pressure to the pulmonary capillaries, creating an imbalance in capillary Starling forces. These alterations increase the regular fluid filtration rate to the interstitium, causing lung stiffness and dyspnea in some patients. The lymphatic system regularly drains the interstitial fluid; however, when the interstitial pressure exceeds the pleural pressure and surpasses drainage capacity, the fluid is directed toward pleural and intra-alveolar spaces, causing pleural effusion and alveolar edema [20].

The pathophysiology of lung congestion differs according to the etiology of CS (i.e., pure acute-onset [ACS, AHF, and myocarditis] or exacerbation of chronic HF [acute on chronic]). The guideline established by the European Society of Cardiology proposed several phenotypes of AHF [11]. The concepts of pure acute-onset HF and exacerbation of chronic HF are occasionally referred to as “vascular failure” and “cardiac failure”, respectively. Vascular failure is defined as a sudden increase in vascular stiffness that leads to a transient volume shift from the peripheral veins to the pulmonary circulation, accompanied by slight fluid accumulation [21]. Cardiac failure is defined as the deterioration of cardiac performance over a period of days to weeks, leading to decompensation with pulmonary edema. This is characterized by the development of symptoms gradually over days and labeled as “normotensive–hypotensive” HF [22]. The mechanisms underlying lung congestion might differ depending on the type of CS (i.e., pure acute-onset or natural progression of advanced HF/exacerbation of chronic HF); hence, the treatment strategy against congestion should be carefully determined based on the profile of each patient.

Ventilator mechanical support after endotracheal intubation (i.e., invasive mechanical ventilation) was the mainstay of respiratory support in patients with AHF up to the 2000s. Since 2010s, almost all patients with AHF have been treated with noninvasive ventilation (NIV) [23]. NIV reduces respiratory distress and improves metabolic disturbances in acute cardiogenic pulmonary edema; therefore, it is strongly recommended as the first option for the management of respiratory failure in AHF [24, 25]. However, NIV might not be feasible in patients with CS due to the high metabolic demand from increased breathing effort, altered mental state resulting in poor synchrony, concomitant cardiac arrest, and severity of pulmonary edema with poor diuretic response causing insufficient oxygenation. These conditions require tracheal intubation and the use of invasive mechanical ventilation [26]. In patients requiring invasive ventilation, lung-protective ventilation (6 mL/kg/body weight tidal volume) should be considered for the prevention of lung injury [27]. The decision to initiate mechanical ventilatory support is multifactorial and mainly depends on arterial blood gas levels, neurologic status, and required interventions. Typically, the resolution of congestion and pleural effusion requires a prolonged period of time in patients with CS versus those with “pure” AHF. Thus, the prolonged use of NIV should be avoided for the prevention of respiration-associated pneumonia. Furthermore, the sputum-expelling ability of patients should be evaluated in detail. Short-term use of NIV is suggested for patients in whom rapid resolution of congestion is expected through appropriate treatment for CS. In patients experiencing exacerbating situations (e.g., increased breathing effort, decreased breathing efficiency and delirium) regardless of the preservation of oxygenation by NIV, physicians should not hesitate to perform tracheal intubation and use invasive mechanical ventilation. The use of a high-flow nasal cannula for respiratory support in patients with AHF was recently proposed as a short-term option [28, 29]. It might constitute another option for the treatment of respiratory failure caused by pleural effusion and/or slight congestion in CS. Although this approach did not sufficiently support positive end-expiratory pressure (target 3–4 mm Hg), it offered an advantage in terms of humidification and assisted in the removal of sputum compared with NIV.

Use of diuretics is recommended as first-line therapy for patients with cardiac failure. In such patients, congestion is attributable to fluid accumulation and volume overload. Administration of furosemide have been used for long time as the first choice of diuretics before evidence-based medicine was established. Carperitide (i.e., human atrial natriuretic peptide) was also used during acute phase in 2000s [30]. Immediate oral administration of tolvaptan has been recommended for the prevention of worsening renal function in AHF [31]. More recently, tolvaptan has been intravenously administered during the acute phase of the disease in Japan [32, 33]. Vasodilators are the first choice for the treatment of vascular failure. Meanwhile, physicians should exercise caution when considering aggressive treatment with vasodilators in patients with CS. The administration of nicorandil, which can decrease PCWP and simultaneously avoid the development of systolic hypertension, might be recommended in such cases [34]. Stabilization of hemodynamics is necessary for the resolution of congestion. Thus, evaluation of CI is also important in this setting. In patients with severely reduced cardiac output, the use of inotropes, inodilators, and vasopressors should be carefully considered, together with diuretics.

### Kidneys

Acute kidney injury (AKI) complicating CS is a well-described phenomenon. Cardiorenal syndrome refers to the pathophysiological interplay between the heart and kidneys. Type 1 cardiorenal syndrome is manifested as an acute cardiac event (e.g., AMI, myocarditis, and AHF) that results in kidney injury and renal dysfunction; this condition is termed AKI in CS [35]. Approximately one-third of patients with CS develop AKI, and they have poor prognosis [36]. CS survivors achieve gradual renal recovery within 5–20 days; however, the recovery period depends on the severity of AKI [37]. Substantial evidence exists for the conclusion that complex and multifactorial mechanisms underlie renal dysfunction in patients with CS, involving both hemodynamic (renal arterial hypoperfusion and renal venous congestion) and non-hemodynamic factors [35, 38, 39]. Non-hemodynamic contributors to AKI in CS include inflammatory mediators (e.g., infection, tissue damage), iatrogenic damage (e.g., contrast media, nephrotoxic medication), and elevated intra-abdominal pressure [40]. Prompt administration of continuous renal replacement therapy (CRRT) may reverse these effects. Therefore, immediate initiation of CRRT is recommended in patients with severe AKI (i.e., creatinine levels more than twofold higher than the baseline levels [41]) and/or low urine output. Approximately 3–6% of patients with CS develop AKI requiring hemodialysis; their in-hospital mortality rate is worse than that of other patients [42, 43].

AKI has been traditionally attributed to the hypoperfusion of the kidneys through progressive impairment of the cardiac output or intravascular volume depletion secondary to CS [36]. However, attention has shifted from cardiac output (“forward failure”) to venous congestion (“backward failure”) as the most important hemodynamic determinant [44]. The development of “congestive kidney failure” induced by the increased renal venous pressure arising from venous congestion (increased renal after-load) and increased renal interstitial pressure (intrinsic renal compromise) might play an important role in the development of AKI in patients with CS [45]. Recently, it was reported that persistent venous congestion, as well as arterial and organ hypoperfusion reflected by a lower arterial pressure and CI, are associated with both the incidence and severity of AKI [36]. Pressure-induced reduction in renal blood flow, renal hypoxia, and increased interstitial fibrosis directly lead to AKI in CS through renal congestion.

The components of AKI were recently introduced in the field of intensive care. Approximately 30% of patients with AHF present with AKI at the time of admission to the intensive-care unit (ICU) [46]. This complication is strongly associated with a high in-hospital mortality rate and poor long-term prognosis [38]. AKI leads to adverse outcomes in patients with AHF [38, 41, 46]. It has been suggested that the presence of AKI on admission is an important factor influencing the outcomes of patients with AHF. Although worsening renal failure (WRF) was also traditionally suggested for the evaluation of renal function, it might be insufficient for the evaluation of AKI. Thus, the combined use of WRF and AKI has been suggested [47]. In a previous study, the presence of AKI on admission and an increase in serum creatinine levels by ≥ 0.3 mg/dL during the first 5 days compared with the baseline levels were independent predictors of long-term mortality [47]. A four-group comparison (i.e., AKI/no-AKI and WRF/no-WRF) was performed in that study [47]. Based on the findings, it was concluded that the presence of WRF with AKI at the time of admission might be defined as true-WRF. Acute renal impairment occasionally develops in patients with AHF, and the involvement of some mechanisms has been suggested. Based on the cardiorenal syndrome, acute renal impairment in AHF may simply be a marker of more severe HF rather than WRF. Further studies are warranted to confirm these findings in patients with CS.

The hemodynamic approach is a primary concern for the treatment of AKI in CS. The therapeutic goal for severe AMI is to achieve the lowest venous filling pressures without deterioration of the cardiac output. As described earlier, the clinical presentation of AKI may be heterogeneous and the degree of renal impairment varies; hence, individualized treatment strategies are required for each patient and phenotype. Diuretics, vasodilators, and inotropes are the mainstay of treatment for AKI in CS, whereas the use of nephrotoxic agents and procedures should be avoided. As an organ-specific therapy, initiation and timing of CRRT for AKI has been evaluated; however, no definitive approach has yet been suggested [48], and the prognostic benefit of CRRT has not yet been observed in real world data [42, 43]. The optimal timing for the initiation/discontinuation, duration, and dose of CRRT to improve prognosis remain controversial as well. CRRT is generally initiated following the occurrence of life-threatening alterations in fluid, electrolyte, and acid–base balance [48]. The initiation of CRRT in patients in whom decongestion has not been achieved by pharmacological treatment is controversial [36]. The timing of CRRT initiation is also debated, and a consensus has not been reached. Currently available data are inconclusive regarding the effectiveness of CRRT versus monotherapy with diuretics. Hence, further investigation is warranted in this field. Finally, renal protection through the use of Impella was recently suggested [49]. The investigators concluded that the targeted control of the renal resistive index, a well-established parameter for the early detection of AKI, could mediate renal organ protection through the initiation of Impella support. Further investigation should closely focus on the effectiveness of Impella support in protecting against the development of AKI.

### Liver

Liver injury is frequently observed in CS. A quarter of patients have an abnormal liver function test in the acute phase, and liver abnormality has been independently associated with mid-term mortality [50]. The levels of aminotransferases peak between 1 and 3 days after hemodynamic collapse, and return to within the normal range after 7–10 days. Patients with AHF occasionally suffer from liver damage, such as congestive liver and liver hypoperfusion, due to low cardiac output [51, 52]. Acute abnormal liver function test results were also observed in patients with these conditions. Hepatic cell injury is generally determined based on the elevation of transaminases, which are markedly and sharply increased in hypoxic hepatitis. The absolute levels of transaminases are associated with a worse in-hospital mortality rate and can be used as biomarkers of hemodynamic reserve [53]. In addition, cholestasis is determined based on the elevation of bilirubin or alkaline phosphatase. Congestive hepatopathy is commonly noted in patients with high venous pressure, particularly in CS patients with right ventricular dysfunction. This condition is accompanied by high levels of direct bilirubin, gamma-glutamyl transferase, and alkaline phosphatase. In patients with chronic HF, a low CI (forward failure) and high CVP (backward failure) were correlated with total bilirubin levels [54, 55]. Previous studies indicated that reduced perfusion and venous congestion are the main causes of elevation in serum total bilirubin levels. This evidence supports the use of serum bilirubin levels in the evaluation of patients with AHF. In the setting of CS, abnormal liver function test results would be observed under both reduced perfusion and venous congestion. These abnormal liver function test results might depend on both conditions, since acute liver dysfunction may not be monocausal. These abnormalities often coexist in CS and are attributed to a combination of both congestion and reduced CI. Hypoxic liver injury (i.e., ischemic hepatitis) might be the most common cause of massive elevations in aminotransferase levels in patients with CS. It represents the diffuse hepatic injury caused by a sudden deterioration of cardiac output, and is accompanied by a sharp elevation in the liver function test results and lactic dehydrogenase levels. It occurs in 5–10% of patients with critical illness, and is an important risk factor for mortality in the ICU [53, 56].

In the absence of specific therapies for liver injury in CS, particular attention must be paid to the improvement of the hemodynamic profile, including reduction in pulmonary vascular resistance and right atrial pressure [50]. Liver-specific management involves appropriate supportive treatment for the conditions induced by liver impairment or failure (e.g., coagulopathy, hypoglycemia, hypoalbuminemia, hepatopulmonary syndrome) [57, 58]. However, these are not specific treatments for patients with CS.

### Intestines

Acute hypoperfusion in CS can compromise the barrier and absorptive functions of the intestines; this clinical situation is termed cardiointestinal syndrome [59]. Abdominal congestion (i.e., splanchnic venous and interstitial congestion) also develops in a substantial number of patients with advanced congestive HF. In CS, hemodynamics (either or both hypoperfusion and congestion) vary considerably between cases. Therefore, the morphology, permeability, and function of the intestines, as well as the growth and composition of intestinal microbiota, may be altered. It is well-recognized that intestinal morphology, permeability, and absorption are altered in chronic HF [60, 61]. Systemic and/or venous congestion, sympathetic vasoconstriction, and sudden deterioration of cardiac output contribute to decreased flow in the splanchnic microcirculation and increase the risk for bowel ischemia. Under these circumstances, ischemia easily develops in the distal intestinal villus due to the organization of the capillary network. Within the intestinal villus, the tip of the structure is vulnerable to hypoxia due to the creation of an area of low oxygen pressure by the countercurrent microcirculatory system [62].

These changes can disrupt the barrier function of the intestines and exacerbate systemic inflammation via microbial or endotoxin translocation into the systemic circulation. Microcirculatory injury in the intestinal barrier leads to increased bacterial translocation [63]. Increased intestinal permeability and an augmented bacterial biolayer may contribute to the development of both chronic inflammation and malnutrition [60, 61]. Ischemia causes epithelial cell dysfunction and loss of the barrier function of the intestines, which allows lipopolysaccharides or endotoxins produced by Gram-negative intestinal bacteria to enter the circulatory system [63]. This entry contributes to cytokine generation and systemic inflammation, leading to several abnormalities of cardiomyocyte function and energetics.

It has been reported that approximately 20% of patients with CS develop a systemic inflammatory response (SIRS) after the initial phase [64]. The cause of infection is not clearly documented in some of those patients. The development of SIRS during the latter phase of CS is independently associated with poor outcomes [64]. Multiple components might contribute to the emergence of SIRS during CS. It is likely that organ injury during the initial phase of CS might lead to an unabated inflammatory reaction. An alteration in the barrier function of the intestines might result in the translocation of bacteria and endotoxins, thereby contributing to this phenomenon.

Few interventions target intestinal protection in these condition of CS [65]. In addition to loop diuretics and vasodilators, paracentesis in patients with ascites and elevated intra-abdominal pressure (> 8 mmHg), or ultrafiltration may be therapeutic strategies to consider in specific patients. Since intestinal-specific treatment may not be possible because of the mechanisms involved, considering the treatment of patients with SIRS (e.g., low doses of corticosteroids and polymyxin B-immobilized fiber column direct hemoperfusion) would be reasonable.

### Brain

Neurogenic outcome is important during the initial treatment of CS. Brain dysfunction and injury (e.g., cerebral infarction, bleeding, and anoxic brain damage) are frequently observed in the initial phase. Cerebral dysfunction is associated with an independent increase in mortality in patients with CS. Cerebral infarction is caused by various conditions, such as hypoperfusion, left ventricular and/or arterial thrombosis, and aortic atherosclerosis. Almost all patients receive treatment with anticoagulation and antiplatelet therapy, which occasionally induces cerebral bleeding. Moreover, the use of mechanical support (ECMO, IABP, and Impella) increases the incidence of these cerebral complications [66]. Anoxic brain damage occurs in approximately 10% of patients with CS, and is associated with prolonged hospital stay and a delay in the return of these individuals to work [67]. Many of these patients also expire following the withdrawal of life-sustaining therapies. Anoxic brain damage is a major concern in a special category of patients with CS, i.e., those who experience out-of-hospital cardiac arrest. Following cardiac arrest, targeted temperature management reduces the overall metabolic rate and myocardial oxygen consumption, thereby contributing to neurological protection [68]. However, there are limited data on CS following cardiac arrest. In the SHOCK-COOL trial, mild therapeutic hypothermia failed to show a substantial beneficial effect on cardiac power index at 24 h in patients with CS after AMI [69]. Another trial involving patients with CS receiving VA-ECMO is currently ongoing, and will investigate the effects of moderate hypothermia on organ function [70].

Higher cortical dysfunction can occur in at least three closely related phenotypes, namely, depression, cognitive dysfunction, and delirium after the initial treatment of CS. In patients with AHF who require intensive care, there was also considerable overlap in impairment among domains of memory, processing speed, executive function; one-third and > 15% of patients experienced impairment in two and three domains, respectively [71]. Shock is the primary cause of delirium particularly in elderly patients; however, multiple other factors (e.g., inflammation, stress, and neurohormonal dysregulation) may also play significant roles [72, 73]. Delirium is independently associated with the adverse outcome (e.g., prolonged ICU stay increased in-hospital mortality) in CS [73, 74]. It is an acute disorder of inattention and global cognitive dysfunction, which is associated with adverse outcomes (prolonged hospital stay) in patients with CS requiring intensive care [73]. Freedom from the infusion line might also help prevent delirium in elderly patients [73].

## Conclusions

CS is a life-threatening disorder associated with high rates of mortality and morbidity. Most deaths in patients with CS occur within the first few days after presentation; therefore, rapid appropriate treatment is essential for patient survival. Hemodynamic treatment strategies through mechanical support and medication have already been established in CS. However, in addition to cardiological approach, it might need to develop organ-specific treatment strategies for patients with CS. Thus, further investigation in large hospitals with ICU is warranted.

## Data Availability

Data sharing not applicable to this article as no data sets were generated or analysed during the current study.

## Abbreviations

CS: Cardiogenic shock
ACS: Acute coronary syndrome
AHF: Acute heart failure
CI: Cardiac index
PCWP: Pulmonary capillary wedge pressure
AMI: Acute myocardial infarction
BNP: B-type natriuretic peptide
IABP: Intra-aortic balloon pump
VA-ECMO: Venoarterial extracorporeal membrane oxygenation
CVP: Central venous pressure
CI: Cardiac index
NIV: Noninvasive ventilation
AKI: Acute kidney injury
CRRT: Continuous renal replacement therapy
WRF: Worsening renal failure
ICU: Intensive care unit
SIRS: Systemic inflammatory response
